# Development and validation of an early prediction model for hypertriglyceridaemic severe acute pancreatitis: a retrospective study

**DOI:** 10.7717/peerj.20607

**Published:** 2026-01-20

**Authors:** Yuzhi Cao, Wenxiu Li, Peng Peng, Jinrong Wu, Xiao Xiao, Xiaoqiang Wan, Cheng He, Chuanming Li, Yongchao Wang, Dianliang Fang

**Affiliations:** 1 Department of Gastroenterology, Chongqing Emergency Medical Center, Chongqing Key Laboratory of Emergency Medicine, Chongqing, China; 2 Department of Radiology, Chongqing Emergency Medical Center, Chongqing Key Laboratory of Emergency Medicine, Chongqing, China; 3 Department of Laboratory, Chongqing Emergency Medical Center, Chongqing Key Laboratory of Emergency Medicine, Chongqing, China

**Keywords:** Hypertriglyceridaemic, Acute pancreatitis, Pancreatic steatosis, Severity, Prediction model, Independent predictors, Pancreatic necrosis, Pleural effusion, Decision curve analysis (DCA), Multivariate logistic regression

## Abstract

**Background:**

The incidence rate of hypertriglyceridaemic acute pancreatitis (HTG-AP) has been steadily increasing due to changes in lifestyle and dietary patterns. Moreover, HTG-AP tends to be more severe than pancreatitis caused by other aetiologies, which may be related to pancreatic steatosis (PS). However, currently, no universally accepted multifactorial clinical scoring system specifically for predicting the severity of HTG-AP exists. This study aimed to identify predictors of hypertriglyceridaemic severe acute pancreatitis (HTG-SAP) and specifically incorporated PS into a visual model for predicting HTG-SAP early.

**Methods:**

A total of 346 patients with HTG-AP were included. These patients were classified into HTG-SAP (*n* = 94) and hypertriglyceridaemic non-severe acute pancreatitis (HTG-NSAP, *n* = 252) groups. An additional 51 patients were included for prospective internal validation of the predictive model. SPSS 29.0 and R version 4.4 software programs were used for statistical data analysis and for establishing and validating the predictive model, employing various methods, including univariate analysis, binary logistic regression, calibration curve analysis, and decision curve analysis (DCA).

**Results:**

Eight variables, namely, respiratory rate (RR), D-dimer (D-D), blood urea nitrogen (BUN), serum calcium (Ca^2+^), potential of hydrogen (pH), and the presence of pancreatic necrosis (PN), pleural effusion (PE) and PS, were identified as independent predictors for HTG-SAP via multivariate binary logistic analysis. The AUC of the new HTG-SAP model was 0.937 (95% CI [0.908–0.966]), which was greater than those of the modified CT severity index (MCTSI), the Bedside Index for Severity in Acute Pancreatitis (BISAP) score, and the Sequential Organ Failure Assessment (SOFA) score (AUC: 0.832, 0.784, and 0.782, respectively) (*P* < 0.001). The calibration curve strongly aligned the predicted outcomes and the actual observations. DCA indicated that clinical intervention would be beneficial for patients who are predicted to be at risk of developing HTG-SAP.

**Conclusion:**

RR; D-D, BUN, and Ca^2+^ levels; pH, and the presence of PN, PE, and PS are independent predictors of HTG-SAP. The prediction model developed based on these predictors highly consistent and practical for predicting HTG-SAP.

## Introduction

Acute pancreatitis (AP) is a critical inflammatory condition of the pancreas characterized by sudden onset and intense abdominal pain, and is the most prevalent gastrointestinal condition leading to hospitalization (Peery et al., 2012). AP occurs when pancreatic enzymes are activated prematurely, resulting in the digestion of pancreatic tissue and inflammation or necrosis in the pancreas and nearby tissues (Szatmary et al., 2022). According to the Atlanta classification, AP is categorized into mild, moderately severe, and severe acute pancreatitis (SAP) (Banks et al., 2013). Although most patients with AP have a mild and self-limiting form that can be managed with simple fluid replacement therapy, approximately 12–20% develop SAP, which progresses to local and/or systemic complications, including systemic inflammatory response syndrome (SIRS) and organ failure lasting more than 48 h (Banks et al., 2013). The mortality rate for patients with AP is approximately 2% (Gapp et al., 2019; Lee & Papachristou, 2019); however, this rate may increase to 28.3–52% in patients with SAP (Petrov et al., 2010; Párniczky et al., 2016; Schepers et al., 2019).

In recent years, the incidence of hypertriglyceridaemic acute pancreatitis (HTG-AP) has increased significantly (Lai et al., 2024). A retrospective study from China revealed that the incidence of HTG-AP increased approximately 2.6-fold over a decade, increasing from 8.4% in April 2012–March 2013 to 22.3% in April 2020–March 2021 (Lin et al., 2022). Several studies have suggested that patients with HTG-AP tend to experience more severe disease progression and have a greater risk of persistent organ failure than those with other aetiologies do (Lai et al., 2024; Zhou et al., 2024). The mechanism of HTG-AP is characteristically different from those of other aetiologies; the excess free fatty acids (FFAs) caused by HTG and elevated chylomicrons increase plasma viscosity, resulting in ischaemia in pancreatic tissue and inflammation in the pancreas (Kiss et al., 2023). Pancreatic steatosis (PS) is defined as excessive lipid deposition in the pancreas, resulting from diverse causes, including aging and metabolic syndrome-related factors such as diabetes, dyslipidaemia, and obesity (Wagner et al., 2022). Therefore, PS might be a good predictor of hypertriglyceridaemic severe acute pancreatitis (HTG-SAP). In a matched case–control study of 50 AP patients, Ko et al. (2022) reported that an 1% increase in PS was significantly associated with a 30% greater chance of experiencing a first attack of AP. A recent prospective cohort study of 42,599 participants revealed that PS was associated with a greater risk of AP (HR 3.982, 95% CI [2.192–7.234]) and was an independent risk factor for AP (*P* = 0.001) (Dong et al., 2024).

Currently, AP scores are frequently used to predict severity and guide early management in clinical settings, primarily including the Sequential Organ Failure Assessment (SOFA), the Modified CT Severity Index (MCTSI), and the Bedside Index of Severity in Acute Pancreatitis (BISAP), among others (Colvin et al., 2020). Nonetheless, a perfect multifactorial scoring system or biochemical marker for the early evaluation of AP severity has not yet been established (Silva-Vaz et al., 2020). Combining multiple biomarkers for prediction is a straightforward and effective method, especially when new indicators are introduced. However, accurate prediction of HTG-SAP remains challenging (Gliem et al., 2021), and few studies in the literature have explored the relationship between PS and HTG-AP severity. Therefore, we aimed to retrospectively analyse the admission data of patients with HTG-AP within 24 h to further investigate the significance of PS and establish a visual model for the early prediction of HTG-SAP, which can support clinical decision-making to reduce morbidity and mortality.

## Methods

### Study design

In this study, we retrospectively analysed a total of 1,288 patients with AP admitted to Chongqing University Central Hospital in China from January 2019 to December 2023. The Ethics Committee of Chongqing Emergency Medical Center approved this study (Approval No. 202445) and granted a waiver of informed consent.

### Patients

**Figure 1:**
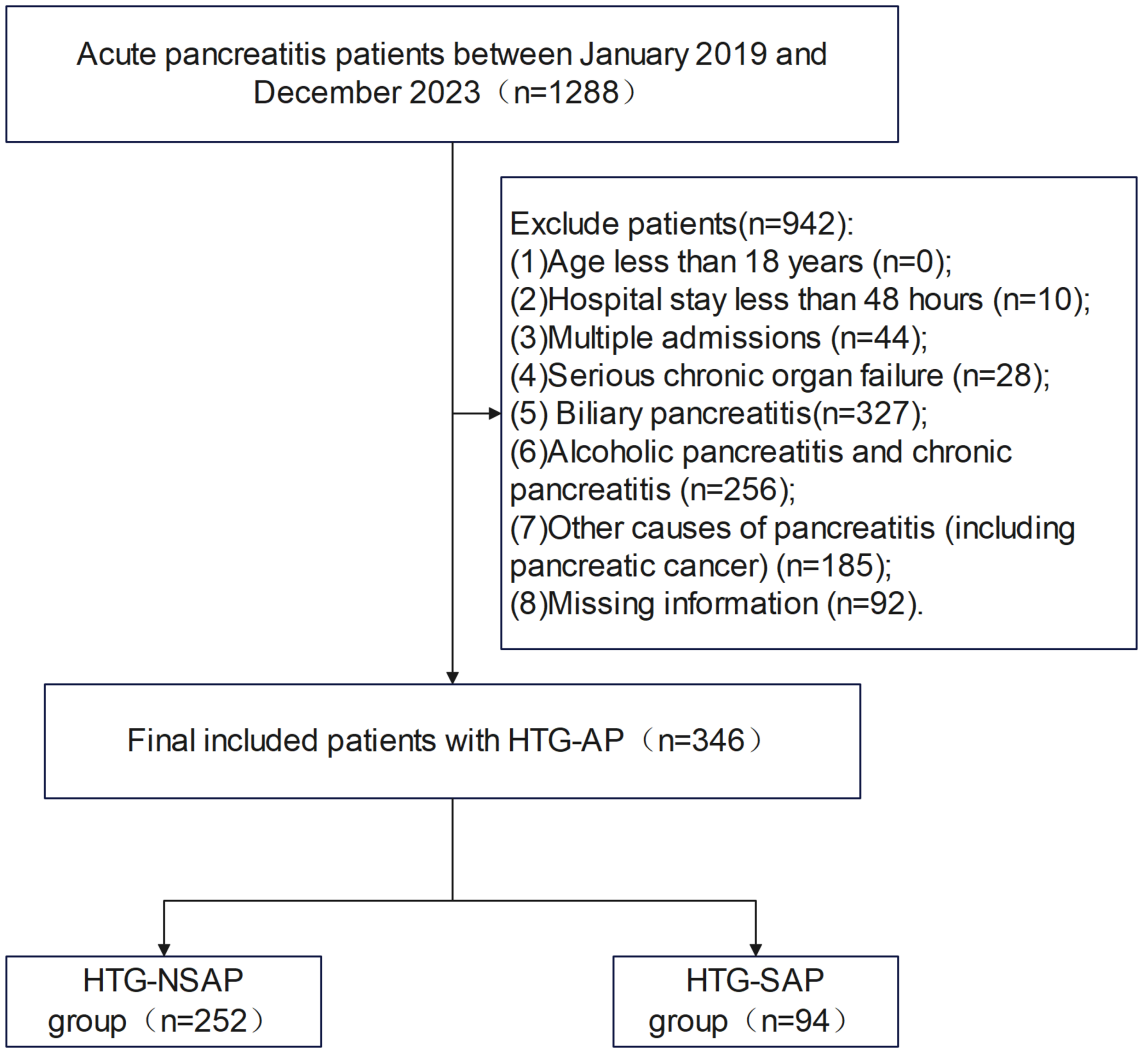
Flowchart of patient selection from Chongqing University Central Hospital. Patients were screened and enrolled according to the predefined inclusion and exclusion criteria, with HTG-SAP (*n* = 94) and HTG-NSAP (*n* = 252).

The inclusion criterion was patients aged ≥18 years with a first-time diagnosis of HTG-AP. The exclusion criteria were as follows: age less than 18 years (*n* = 0), hospital stay less than 48 h (*n* = 10), multiple admissions (*n* = 44), serious chronic organ failure (*n* = 28), biliary pancreatitis (*n* = 327), alcoholic pancreatitis and chronic pancreatitis (*n* = 256), other causes of pancreatitis (including pancreatic cancer) (*n* = 185), and missing information (*n* = 92). Overall, 346 patients with HTG-AP were included in the study. These patients were classified into two groups: those with HTG-SAP (*n* = 94) and those with hypertriglyceridaemic nonsevere acute pancreatitis (HTG-NSAP; *n* = 252). The HTG-NSAP group included patients with mild and moderately severe AP (Banks et al., 2013) (Fig. 1).

### Definitions

AP was diagnosed when any two of the following three characteristics were present: (1) persistent distending pain in the epigastric region; (2) serum lipase and/or amylase activity 3 times higher than the normal upper limit; and (3) any evidence of AP on imaging, including computed tomography (CT), magnetic resonance imaging (MRI), or transabdominal ultrasound (US) (Banks et al., 2013; Mederos, Reber & Girgis, 2021). For the diagnosis of HTG-AP, all of the following criteria had to be met: (1) the established AP diagnostic criteria; (2) a triglyceride (TG) concentration ≥1,000 mg/dl or a TG concentration of 500–1,000 mg/dl with chylous serum; and (3) the exclusion of AP resulting from alternative aetiologies, such as biliary diseases, alcohol, trauma, and tumours, *etc*. (De Pretis, DeMarchi & Frulloni, 2020).

PS, which is characterized by the accumulation of fat in the pancreas, was initially identified by Ogilvie in a cadaver study conducted in the early 20th century (Wagner et al., 2022). The literature reports several synonyms for ‘pancreatic fat accumulation, such as pancreatic lipomatosis, PS, fatty replacement, fatty infiltration, fatty pancreas, lipomatous pseudohypertrophy, and nonalcoholic fatty pancreas disease (NAFPD) (Smits & Van Geenen, 2011), whereas ‘steatosis’ is a broad term referring to the accumulation of intracellular fat within parenchymal cells. PS specifically refers to the accumulation of fat within islet cells or acinar cells (Petrov, 2023).

Delirium,” was diagnosed based on the following criteria: (1) acute onset and fluctuating course of symptoms, (2) inattention, (3) disorganized thinking, and (4) altered level of consciousness, as defined by the Confusion Assessment Method (CAM) (Mattison, 2020). Severe chronic organ failure was defined as the presence of any of the following: Respiratory failure: Type I (PaO_2_ < 60 mm Hg with normal or low PaCO_2_) or Type II (PaO_2_ <  60 mm Hg with PaCO_2_ > 50 mm Hg). Liver failure: Child–Pugh class C or D. Cardiac failure: New York Heart Association (NYHA) class III or IV. Renal failure: Chronic kidney disease (CKD) stage 4 or 5 (Brakenridge, Kornblith & Cuschieri, 2024).

### Clinical assessment of severity

As outlined by the 2012 Revised Atlanta Classification (RAC) (Banks et al., 2013), the severity of AP can be categorized into three groups according to the presence of organ failure and local and/or systemic complications. Mild AP (MAP) was defined as the absence of organ failure and local or systemic complications. Moderately severe AP (MSAP) was characterized by transient organ failure (lasting less than 48 h) and/or the presence of local complications or the exacerbation of preexisting comorbid conditions. Severe AP (SAP) was indicated by organ failure that persisted for more than 48 h. Organ failure was identified by assessing the respiratory, cardiovascular, and renal systems and was defined as a modified Marshall score greater than 2 in any of these three systems. Local complications included acute peripancreatic fluid collection, pancreatic pseudocysts, acute necrotic collection, walled-off necrosis, and infected pancreatic necrosis (PN).

### Assessment of PS by CT

Numerous radiological techniques for assessing PS, including US, endoscopic ultrasound (EUS), CT, and MRI, have been reported in the literature. Among these methods, CT is among the most frequently utilized methods for evaluating PS (Kim et al., 2014; Fukuda et al., 2017; Petrov, 2023). PS was evaluated at baseline by two experienced radiologists by measuring the mean degree of pancreatic attenuation *via* the Hounsfield unit (HU) scale on CT scans using a specialized workstation with the Image Management and Communications System (IMPAX6.5.3 AGFA Healthcare N.V. from Belgium). The comprehensive details of all the patients were concealed from the two experienced radiologists to prevent bias. Pancreatic attenuation was assessed in the pancreatic parenchyma of the head, body, and tail by placing three round regions of interest (ROIs) of 1.0 cm^2^ in each section. The round ROIs were placed away from blood vessels, ducts, calcifications, and any areas of necrosis to ensure measurement of healthy pancreatic parenchyma to minimize bias. The degree of pancreatic attenuation was subsequently determined by calculating the average CT number values from the nine ROIs for each patient. Similarly, the spleen and liver attenuation levels were determined by calculating the average CT number values from the three ROIs, each with an area of 1.0 cm^2^, for each patient (Kim et al., 2014; Ko et al., 2022). PS was defined as a pancreas-to-spleen CT number ratio (P/S ratio) of less than 0.70 (Fukuda et al., 2017).

### Data collection

The general characteristics collected for the two groups included age, sex, history of type 2 diabetes, history of hypertension, history of coronary heart disease, temperature (T), heart rate (HR), respiratory rate (RR), mean blood pressure (MBP), and delirium (De). The data from the laboratory tests performed within 24 hours of admission included the following: C-reactive protein (CRP) level, white blood cell (WBC) count, neutrophil (N) count, neutrophil ratio (NR), monocyte (M) count, lymphocyte (L) count, haematocrit (HCT), platelet (PLT) level, activated partial thromboplastin time (APTT), prothrombin time (PT), international normalized ratio of coagulation (INR), fibrinogen (FIB) level, D-dimer (D-D) level, lipase (LPS) level, amylase (AMY) level, procalcitonin (PCT) level, albumin (ALB) level, total bilirubin (TB) level, blood urea nitrogen (BUN) level, serum creatinine (Cr) level, serum calcium (Ca^2+^) level, glucose (GLU) level, lactic acid (LAC) level, total cholesterol (TC) level, (TG level, low-density lipoprotein (LDL) level, potential of hydrogen (pH), partial pressure of carbon dioxide (PaCO2), partial pressure of oxygen (PaO2), and fraction of inspired oxygen (FiO2). The CT imaging data of the abdomen and chest for all patients collected within 24 hours of admission were reviewed. Data on the presence of PN, pleural effusion (PE), abdominal effusion (AE), the CT scan value of the spleen (three measurements), the CT scan value of the liver (three measurements), and the CT scan value of the pancreas (head, body and tail for each of the three measurements) were collected. The mean CT scan values for the liver (MCT_L), spleen (MCT_S), and pancreas (MCT_P) were calculated. PS was defined as a P/S ratio (MCT_P/MCT_S) of less than 0.7 (Fukuda et al., 2017). Additionally, severity scores, including the SOFA, BISAP and MCTSI scores, were also collected or calculated based on the above conditions.

On the basis of previous studies that identified 5–10 potential modelling predictors and considered a 20% HTG-SAP composite outcome from preliminary investigations, we estimated that a sample size of 250–500 patients (including 50–100 patients with the HTG-SAP composite outcome) would be necessary. This estimated number ensures sufficient precision in model construction to comply with the principle of having at least ten outcome events per variable in regression analysis.

### Statistical analysis

Variables conforming to a normal distribution are expressed as the means ± standard deviations, and comparisons between groups were conducted using the independent samples *t* test. Variables with nonnormal distributions are expressed as medians (P25, P75), and comparisons between groups were performed using the Mann–Whitney *U* test. Categorical variables are expressed as counts and percentages (%), and the chi-square test was used for comparisons between groups. Univariate logistic regression analyses were performed to identify variables with statistical significance (*P* < 0.05), which were then included in the multivariate logistic regression (forward stepwise) model. Based on the results of the regression analysis, we identified multiple independent risk factors affecting the severity of AP and constructed a predictive model. Receiver operating characteristic (ROC) curves were generated to assess and compare the areas under the curve (AUCs). ROC curves were generated for the predictive model as well as for the BISAP, MCTSI, and SOFA scores. Additionally, calibration curves were used to assess the agreement between the observed outcomes and predicted probabilities, whereas decision curve analysis (DCA) was employed to evaluate the clinical net benefit of the predictive model. Finally, data collected from an additional 51 patients from January to August 2024 at the same centre were incorporated into the prediction model for prospective internal validation. The interrater reliability for the binary PS classification between the two independent radiologists was assessed using Cohen’s kappa statistic (poor agreement: 0.00; slight agreement: 0.00–0.20; fair agreement: 0.21–0.40; moderate agreement: 0.41–0.60; substantial agreement: 0.61–0.80; almost perfect agreement: 0.81–1.00) (Landis & Koch, 1977). Using a nonparametric comparison of ROC curves (Wilcoxon statistic) *via* the VassarStats online tool (http://vassarstats.net/roc_comp.html), the AUC of our model was compared with those of the BISAP, MCTSI, and SOFA scores to evaluate its predictive performance; exact *p*-values were obtained from this analysis (Hanley & McNeil, 1982). SPSS 29.0 software was used for statistical analyses of the final included data. R 4.4.1 software was used to construct and validate the nomogram model for predicting HTG-SAP. A *P* value of less than 0.05 indicated a statistically significant difference.

## Results

### Univariate analysis results

#### Baseline characteristics

As shown in Fig. 1, a total of 346 patients with AP were included in the final analyses, comprising 94 patients (27.2%) in the SAP group and 252 patients (72.8%) in the non-SAP group. Patients who experienced their first episode of HTG-AP accounted for 26.9% of all patients with AP. There were no significant differences in age, sex, history of hypertension, or history of coronary heart disease between the HTG-NSAP and HTG-SAP groups (*P* > 0.05). However, T, HR, and RR were greater and MBP was significantly lower in the HTG-SAP group than in the HTG-NSAP group (all *P* < 0.05). Additionally, the incidence of MAs was significantly greater in the HTG-SAP group than in the HTG-NSAP group (*P* <  0.05). The MCTSI, BISAP, and SOFA scores were significantly greater in the HTG-SAP group than in the HTG-NSAP group (all *P* < 0.05) (Table 1).

**Table 1:** Baseline characteristics of the HTG-NSAP and HTG-SAP groups. This table shows the baseline characteristics of the HTG-NSAP and HTG-SAP groups, and a univariate analysis was performed, with a *P*-value of <0.05 indicating statistical significance.

Variables	HTG-NSAP (*n* = 252)	HTG-SAP (*n* = 94)	*P* value
Age	40 (34,49)	37 (30,48)	0.094
Sex, *n* (%)			0.263
Male	194 (77.0)	61 (64.9)	
Female	58 (23.0)	33 (35.1)	
History of T2DM, *n* (%)	51 (20.2)	37 (39.4)	<.001
History of HTN, *n* (%)	27 (10.7)	11 (11.7)	0.794
History of CAD, *n* (%)	5 (2.0)	1 (1.0)	0.560
T (°C)	36.7 (36.5,37.0)	36.8 (36.5,37.3)	0.008
HR (frequency/minute)	100 (88,110)	103 (94,129)	0.002
RR (frequency/minute)	20 (20,21)	22 (21,23)	<.001
MBP (mmHg)	105 ± 14	98 ± 19	0.014
De, *n* (%)	1 (0.4)	3 (3.2)	0.031
SIRS, *n* (%)	172 (68.3)	88 (93.6)	<.001
SOFA (score)	1 (0.25,3)	3 (2.75,4)	<.001
MCTSI (score)	2 (2,4)	4 (4,4)	<.001
BISAP (score)	1 (0,3)	3 (2,3)	<.001

**Notes.**

Abbreviations HTG-NSAP hypertriglyceridaemic nonsevere acute pancreatitis HTG-SAP hypertriglyceridaemic severe acute pancreatitis T2DM type 2 diabetes mellitus HTN hypertension CAD coronary heart disease T temperature HR heart rate RR respiratory rate MBP mean blood pressure De delirium SIRS systemic inflammatory response syndrome SOFA Sequential Organ Failure Assessment MCTSI modified CT Severity Index BISAP Bedside Index for Severity in Acute Pancreatitis

#### Clinical characteristics

The interrater reliability for the PS assessment was substantial, with a Cohen’s kappa value of 0.72 (*P* < 0.001). Among patients with HTG-AP, those with PS accounted for 44.2%. There were no significant differences in the WBC count, N count, NR, M count, PLT level, TC level, TG level, LDL level, PaO2, MCT_L, or MCT_S between the HTG-NSAP and HTG-SAP groups (*P* > 0.05). In the HTG-SAP group, the CRP level, HCT, APTT, PT, INR, FIB level, D-D level, LPS level, AMY level, PCT level, TB level, BUN level, Cr level, GLU level, and LAC level were significantly greater than those in the HTG-NSAP group (*P* < 0.05). Conversely, the L count, serum ALB concentration, Ca^2+^, pH, PaO2/FiO2, and MCT_P were significantly lower in the HTG-SAP group than in the HTG-NSAP group (*P* < 0.05).

The proportions of patients with PN, PE, AE, and PS were significantly greater in the HTG-SAP group than in the HTG-NSAP group (*P* < 0.05) (Table 2).

**Table 2:** Clinical characteristics of the HTG-NSAP and HTG-SAP groups. This shows the clinical characteristics of the HTG-NSAP and HTG-SAP groups, and a univariate analysis was performed, with a *P*-value of <0.05 indicating statistical significance.

Variables	HTG-NSAP (*n* = 252)	HTG-SAP (*n* = 94)	*P* value
CRP (mg/L)	25.58 (9.78,81.94)	115.90 (75.26,227.85)	<.001
WBC (×10^9^/L)	13.8 (10.5,16.6)	12.5 (9.5,17.0)	0.317
N (×10^9^/L)	11.58 (8.37,14.48)	10.98 (8.40,14.91)	0.584
NR (%)	86 (79,90)	87 (82,90)	0.134
M (×10^9^/L)	0.55 (0.36,0.75)	0.53 (0.37,0.87)	0.242
L (×10^9^/L)	1.24 (0.90,1.83)	0.89 (0.67,1.30)	<.001
PLT (×10^9^/L)	216 (175,259)	208.00 (158,269)	0.682
HCT (%)	44.15 (40.63,47.00)	46.05 (39.95,50.48)	0.044
APTT (second)	34.05 (31.70,37.40)	36.05 (32.60,39.95)	0.004
PT (second)	12.70 (12.30,13.40)	13.80 (12.50,15.08)	<.001
INR (/)	0.96 (0.91,1.02)	1.05 (0.94,1.18)	<.001
FIB (g/L)	3.73 (3.09,5.21)	4.67 (3.53,7.46)	<.001
D-D (μg/L)	0.76 (0.52,1.15)	2.24 (1.18,4.05)	<.001
LPS (U/L)	416.60 (152.65,940.10)	721.90 (276.88,1483.05)	<.001
AMY (U/L)	227.50 (95.00,572.50)	405.50 (158.00,873.25)	<.001
PCT (ng/L)	0.10 (0.04,0.21)	1.30 (0.23,7.03)	<.001
ALB (g/L)	44.30 (41.03,46.70)	37.45 (33.45,44.38)	<.001
TB (μ mol/L)	12.75 (8.30,17.80)	16.20 (10.50,24.00)	<.001
BUN (mmol/L)	4.21 (3.49,5.32)	5.08 (3.94,8.80)	<.001
Cr (μ mol/L)	54.75 (40.40,67.95)	65.35 (40.43,117.50)	<.001
Ca^2+^ (mmol/L)	2.26 (2.11,2.37)	1.83 (1.42,2.22)	<.001
GLU (mmol/L)	10.64 (7.80,15.47)	16.00 (11.80,21.33)	<.001
LAC (mmol/L)	2.53 (2.00,3.29)	3.38 (2.27,4.98)	<.001
TC (mmol/L)	8.37 (6.46,11.90)	9.18 (6.58,14.27)	0.157
TG (mmol/L)	20.05 (11.84,31.80)	20.99 (10.53,37.80)	0.494
LDL (mmol/L)	1.87 (1.12,2.89)	1.70 (1.02,3.35)	0.479
PH (value)	7.39 (7.35,7.42)	7.34 (7.29,7.38)	<.001
PaCO2 (mmHg)	33 (30,36)	29 (24,34)	<.001
PaO2 (mmHg)	110 (96,130)	105 (88,132)	0.27
PaO2/FiO2 (value)	386 (297,499)	270 (219,336)	<.001
PN, *n* (%)	24 (9.5)	48 (51.1)	<.001
PE, *n* (%)	40 (15.9)	57 (60.6)	<.001
AE, *n* (%)	22 (8.7)	38 (40.4)	<.001
MCT_L (HU)	50.05 (41.52,55.31)	46.92 (39.52,54.74)	0.102
MCT_S (HU)	53.05 (50.73,55.90)	53.15 (49.74,56.14)	0.948
MCT_P (HU)	41.40 (36.09,47.16)	35.69 (33.11,38.43)	<.001
PS, *n* (%)	78 (31.0)	75 (79.8)	<.001

**Notes.**

Abbreviations HTG-NSAP hypertriglyceridaemic nonsevere acute pancreatitis HTG-SAP hypertriglyceridaemic severe acute pancreatitis CRP C-reactive protein WBC white blood cell N neutrophil NR neutrophil ratio M monocyte L lymphocyte HCT haematocrit PLT platelet APTT activated partial thromboplastin time PT prothrombin time INR international normalized ratio of coagulation FIB fibrinogen D-D D-dimer LPS lipase AMY amylase PCT procalcitonin ALB albumin TB total bilirubin BUN blood urea nitrogen Cr creatinine Ca^2+^ calcium GLU glucose LAC lactic acid TC total cholesterol TG triglyceride LDL low-density lipoprotein PH potential of hydrogen PaCO2 partial pressure of carbon dioxide PaO2 partial pressure of oxygen FiO2 fraction of inspired oxygen PN pancreatic necrosis PE Pleural effusion AE abdominal effusion MCT_L mean of CT scan value of the liver MCT_S mean of CT scan value of the spleen MCT_P mean of CT scan value of the pancreas PS pancreatic steatosis PS was defined as a P/S ratio (MCT_P/MCT_S) of <0.7

### Predictors of HTG-SAP and nomogram development

Twenty-seven variables with significant differences between the two groups in the univariate analysis were selected, which included T, HR, RR, De, CRP level, HCT, APTT, PT, INR, FIB level, D-D level, LP level S, AMY level, PCT level, TB level, BUN level, Cr level, GLU level, LAC level, L count, ALB level, Ca^2+^ level, pH, PaO2/FiO2, MCT_P, and the presence of PN, PE, AE and PS (all *P* < 0.05). Ultimately, eight variables, including RR, D-D level, BUN level, Ca^2+^ level, pH level, and the presence of PN, PE and PS, were identified as independent risk factors for HTG-SAP by multivariate binary logistic regression analysis. Among these variables, RR, D-D level, BUN level, and the presence of PN, PE, and PS were independent risk factors, whereas the pH and Ca^2+^ level were independent protective factors (Table 3).

**Table 3:** Results of the multivariate logistic regression analysis of the independent predictors. Eight predictors were identified as independent risk factors for HTG-SAP by multivariate binary logistic regression analysis (*P* < 0.05).

Predictor	*β*	Wald	*P* value	OR	95% CI
RR	0.163	6.384	0.012	1.177	1.037∼1.336
D-D	0.517	13.432	<0.001	1.677	1.272∼2.212
BUN	00.065	6.503	0.011	1.067	1.015∼1.122
Ca^2+^	−0.921	3.955	0.047	0.398	0.161∼0.987
PH	−6.943	8.646	0.003	0.001	0.000∼0.099
PN	1.840	18.415	<0.001	6.298	2.718∼14.596
PE	1.670	17.571	<0.001	5.313	2.433∼11.600
PS	1.546	14.188	<0.001	4.693	2.099∼10.491
Constant	45.130	6.717	0.010	–	–

**Notes.**

Abbreviations *β* Regression coefficient RR Respiratory rate D-D D-dimer BUN Blood urea nitrogen Ca^2+^ Serum calcium PH Potential of hydrogen PN pancreatic necrosis PE Pleural effusion PS Pancreatic steatosis

**Figure 2:**
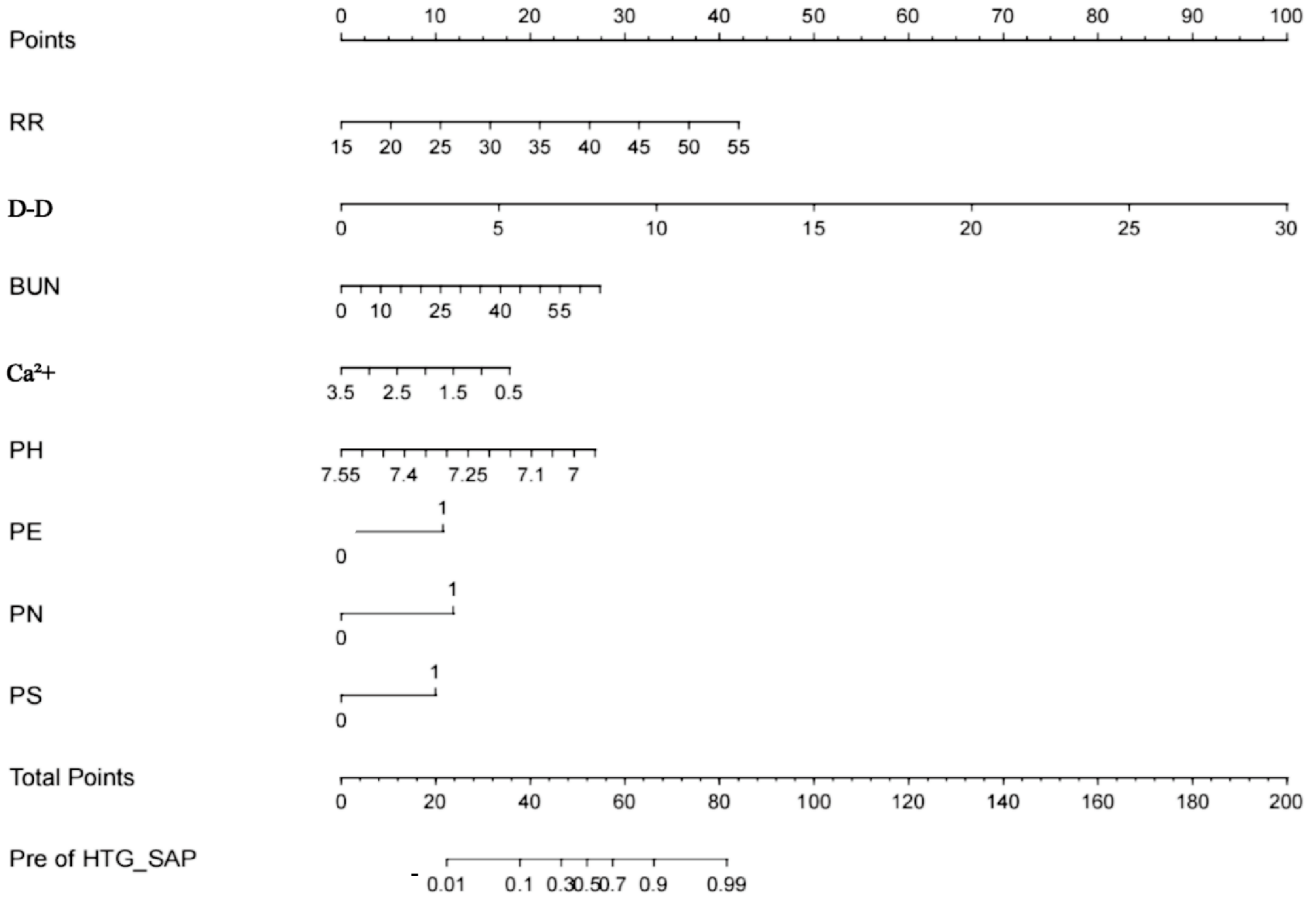
Nomogram for the early prediction model for hypertriglyceridaemic severe acute pancreatitis (HTG-SAP). Points were assigned to patients on the basis of the values of the RR (frequency/minute), D-D level (μg/L), BUN level (mmol/L), Ca 2+ level (mmol/L), pH (value), and the presence of PN, PE, and PS (second to ninth lines) by finding the appropriate points on the ‘RR’, ‘D-D’, ‘BUN’, ‘Ca 2+’, ‘pH’, ‘PN’, ‘PE’ and ‘PS’ scales and then projecting a vertical line upwards to the ‘Points’ scale at the top of the nomogram. These points were subsequently summed , and the corresponding score on the “Total Points” scale is shown. Finally, a vertical line was projected downwards from the ‘Total Points’ scale to the ‘Pre of HTG-SAP’ scale to determine each patient’s predicted risk.”.

In accordance with the results of the multivariate analysis, the HTG-SAP prediction model was established as a nomogram based on RR, D-D level, BUN level, Ca^2+^ level, pH, and the presence of PN, PE and PS, as shown in Fig. 2.

### Validation of the HTG-SAP prediction model

**Figure 3:**
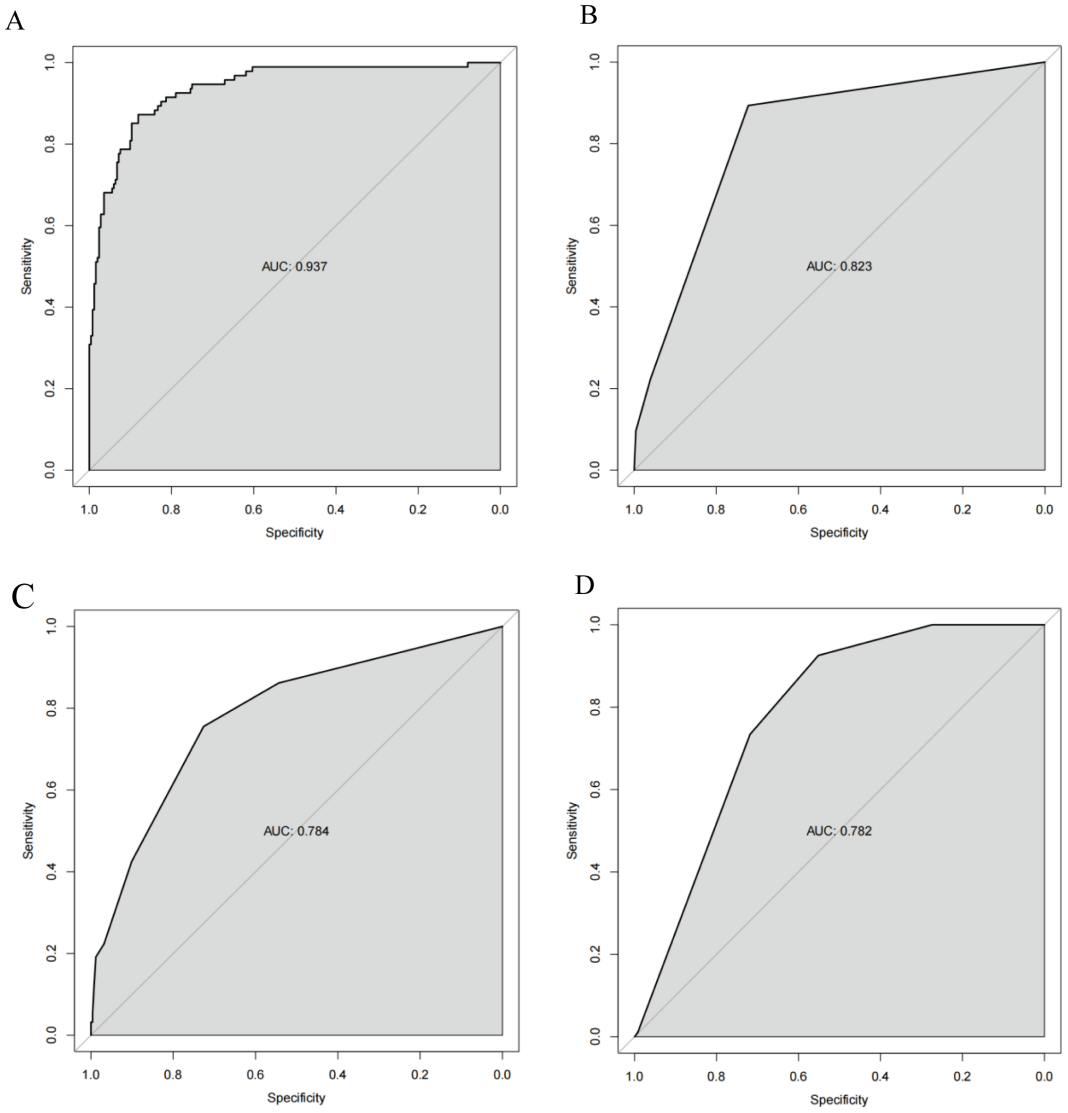
ROC curve of the nomogram for predicting HTG-SAP. The shaded area represents the AUC value. (A) The AUC of the Pre-HTG-SAP model for the prediction of HTG-SAP was 0.937. (B) The AUC of the MCTSI score for the prediction of HTG-SAP was 0.823. (C) The AUC of the SOFA score for the prediction of HTG-SAP was 0.784. (D) The AUC of the BISAP score for the prediction of HTG-SAP was 0.782.

**Figure 4:**
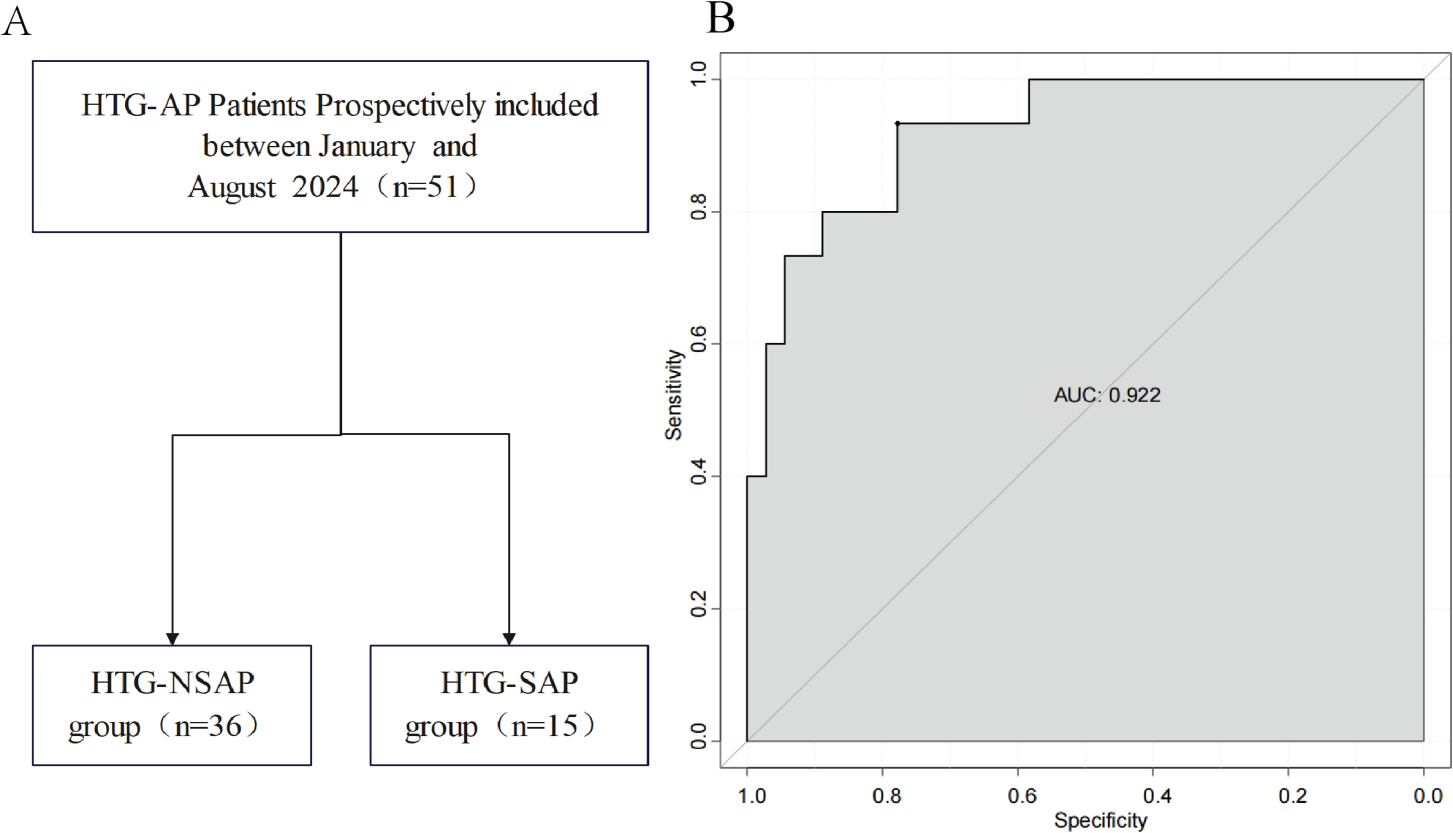
Prospective internal validation of the prediction model. The shaded area represents the AUC value. (A) Flowchart of patient selection; (B) ROC curve.

**Figure 5:**
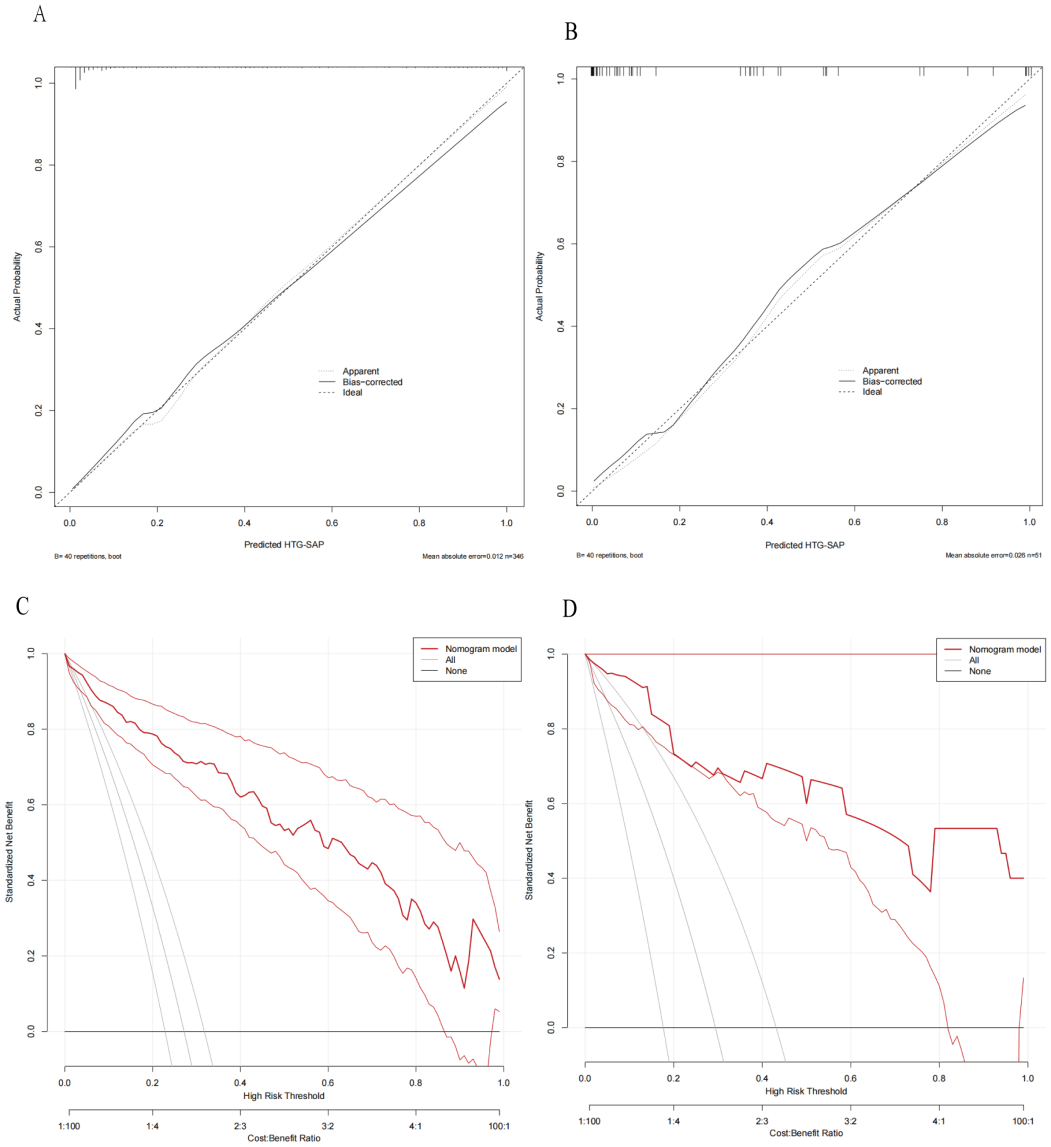
Calibration curves and DCA of the nomogram for predicting HTG-SAP. Calibration curves of the predicted nomogram in the training set (A) and validation set (B); DCA of the nomogram in the training set (C) and the validation set (D). In both (A) and (B), the calibration curves (Apparent and Bias-correctd) demonstrated close proximity to the ideal prediction line (thick dashed line), indicating excellent agreement between the model’s predicted probabilities and actual observed probabilities, thus confirming the model’s predictive accuracy. In both (C) and (D), the net benefit values of the model (“Nommogram model” line, red curves) consistently exceeded the benefit values of intervening for all individuals (‘All’ line, solid gray line), demonstrating the model’s clinical utility.

The ROC curves revealed that the ability of the new model to predict the progression of patients to HTG-SAP was better than those of the MCTSI, SOFA, and BISAP scores. The AUC values of the MCTSI, SOFA, BISAP scores and the HTG-SAP prediction model were 0.832, 0.784, 0.782 and 0.937, respectively, and the differences were all significant (*P* < 0.001) (Table 4, Fig. 3). The results of the ROC analyses indicated that the nomogram strongly predicted patients with HTG-SAP in the training (AUC = 0.937) and validation (AUC = 0.922) cohorts (Fig. 4). The calibration curve demonstrated that the predicted probabilities closely matched the observed outcomes in both the training and validation cohorts, indicating successful calibration (Fig. 5). According to the DCA, the nomogram provided a superior overall net benefit across a broad range of threshold probabilities, highlighting its strong potential for clinical utility (Fig. 5).

**Table 4:** Comparison of the AUCs between the Pre-HTG-SAP and prediction systems.

Model	*P* value (Model *VS.* pre-HTG-SAP)
MCTSI	<0.001
SOFA	<0.001
BISAP	<0.001

**Notes.**

We used a web tool to perform the comparison between two AUCs (nonparametric Wilcoxon statistic) http://vassarstats.net/roc_comp.html).

Abbreviations AUC Area under the curve MCTSI Modified CT Severity Index BISAP Bedside Index for Severity in Acute Pancreatitis SOFA Sequential Organ Failure Assessment Pre-HTG-SAP Predictive model for hypertriglyceridaemic severe acute pancreatitis

## Discussion

In this study, we found that the incidence of the first occurrence of HTG-AP accounted for 26.9% of all cases of AP, which is higher than that of alcohol-induced AP, previously reported as the second leading cause of AP (Yu et al., 2019; Lin et al., 2022). A recent retrospective study including 10,071 cases of AP reported that hyperlipidaemia (25.3%) was the second most common aetiology after cholelithiasis (Lai et al., 2024). In Taiwan area, the incidence of HTG-AP was reported to range from 6.3–12.3% (Olesen et al., 2021). Additionally, the incidence rate of HTG-SAP among patients with HTG-AP was reported to be 27.2%, which is higher than the previously reported incidence rate of SAP among all patients with AP (10–20%) (Banks et al., 2013). This may be related to the unique pathogenesis and pathophysiological processes of HTG-AP. We also revealed that PS is an independent predictor of HTG-SAP, and the incidence of PS in patients with HTG-AP is 44.2%, which is higher than the rates reported in the general population (16–35%) (Wong et al., 2014; Lesmana et al., 2015; Koç & Taydaş, 2020). Furthermore, we developed a simplified and practical nomogram model to predict HTG-SAP with high accuracy (AUC= 0.937) based on independent predictors, including the RR, D-D level, BUN level, Ca^2+^ level, pH, and the presence of PN, PE and PS, within 24 h of admission. To validate the consistency and clinical applicability of our HTG-SAP prediction model, we compared our model with three well-established AP prognostic scoring systems. The results confirmed the accuracy of the model in predicting the occurrence of HTG-SAP.

The pathogenesis of HTG-AP is currently recognized as follows: (1) Excessive TGs in the blood are broken down into FFAs by pancreatic lipase. When the concentration of FFAs exceeds the binding capacity of ALB, protein kinases are activated, resulting in autodigestion of pancreatic cells. (2) FFAs trigger inflammatory responses, causing cytotoxicity and damage to pancreatic capillaries. (3) As blood viscosity increases, serum lipoprotein particles accumulate, potentially leading to embolization of pancreatic vessels. This exacerbates pancreatic ischaemia and acidosis, further enhancing the lipotoxic effects of FFAs (Kiss et al., 2023). Our study revealed that the presence of PS is a novel independent predictor of HTG-SAP. PS indicates the accumulation of pancreatic fat cells (Kiss et al., 2023). Lipid accumulation triggers cellular stress responses, lipotoxic effects, and dysfunctional intercellular signalling, leading to inflammation within the pancreatic microenvironment (Petrov, 2023), which forms the foundation for the onset and progression of HTG-AP. PS acts as a mechanistic pathological driver for most nongenetic pancreatic disorders. Dysfunction of intrapancreatic adipocytes and associated lipotoxicity can surpass an individual’s threshold, triggering inflammation within the pancreatic microenvironment and leading to cellular injury. The resulting inflammatory milieu is intensified by various forms of cellular injury and death, such as ferroptosis and pyroptosis. A critical mechanism in initiating acinar cell injury is the sustained aberrant interplay between lipid droplets and the endoplasmic reticulum. This interaction leads to the release of nonesterified fatty acids, cytosolic and mitochondrial calcium overload, mitochondrial dysfunction, and a compromised autophagic response, which collectively play major roles in the onset of acute pancreatitis (Petrov, 2023). The notably higher severity observed in HTG-AP can be primarily attributed to the lipotoxicity mediated by excessive FFAs. This concept is robustly supported by experimental evidence showing that the hydrolysis of triglycerides within the pancreas leads to a direct, dose-dependent toxic insult, resulting in oedema, haemorrhage and profound acinar cell injury (Saharia et al., 1977; Durgampudi et al., 2014; De Oliveira et al., 2019). This primary lipotoxic assault is further compounded by a secondary phenomenon of plasma hyperviscosity (Saharia et al., 1977). The elevated triglyceride levels increase blood viscosity, impairing pancreatic microcirculation and leading to ischaemia. The resulting cellular acidosis then creates a permissive environment for the intracellular activation of trypsinogen, thereby synergistically amplifying the initial inflammatory and autodigestive processes (Sakorafas, Tsiotos & Sarr, 2000). Furthermore, PS, which is characterized by the accumulation of intrapancreatic adipocytes, is closely linked to more severe presentations of acute pancreatitis (Petrov et al., 2010). The pathophysiological role of PS is likely mediated through adipocyte dysfunction and consequent lipotoxicity, driving a more intense inflammatory response and tissue damage. Collectively, the mechanisms of lipotoxicity and hyperviscosity in HTG-AP, often exacerbated by the presence of PS, provide a compelling explanation for the aggressive clinical course observed in these patients. A Mendelian randomization study revealed that genetically predicted PS was significantly associated with AP (OR per 1-SD increase: 1.40 95% CI [1.12–1.76], *p* = 0.0032), supporting a causal role of PS in pancreatitis (Yamazaki et al., 2024). Another study by Xie et al. (2019) which included 246 patients with AP, further demonstrated that PS was independently correlated with severity, mortality, and systemic complications in patients with AP, which is similar to our results. However, to date, no studies have investigated the relationship between PS and the severity of HTG-AP in clinical practice. Our research addresses this gap in the literature and indicates that PS is an independent predictor of HTG-SAP.

According to the RAC (Banks et al., 2013), organ failure is determined by evaluating three organ systems during the progression of AP: the respiratory, cardiovascular, and renal systems. In our study, RR, BUN level and presence of PE were confirmed to be independent predictors of HTG-SAP. The lipotoxicity from FFAs generated from TGs is among the main theories explaining how HTG-AP is aggravated (Kiss et al., 2023). These toxic structures from FFAs can damage platelets, the vascular endothelium, and acinar cells, leading to ischaemia and acidosis; acidosis further enhances FFA toxicity by activating trypsinogen. This direct damage can lead to pancreatic duct rupture and increased capillary permeability throughout the body (Kiss et al., 2023). Additionally, FFAs may directly stimulate the production of inflammatory mediators, including TNF-alpha, IL-6, and IL-10, thereby intensifying the local inflammatory response and initiating the inflammatory cascade (De Pretis, Amodio & Frulloni, 2018). These inflammatory mediators travel through the lymphatic and systemic circulation, where they reach the liver, lungs, heart, kidneys, and gastrointestinal tract, resulting in SIRS (Szatmary et al., 2022). Based on the above pathophysiological processes, patients with HTG-AP may develop lung injury, kidney injury, hypoproteinaemia and third-space fluid accumulation. These processes are manifested by an increased RR, an elevated BUN level, and the presence of PE. A retrospective study of 326 elderly patients indicated that RR is an important predictor of the survival of patients with SAP (Zhu et al., 2024). The increase in BUN levels in patients with HTG-AP can be explained by two main mechanisms of acute kidney injury (Nassar & Qunibi, 2019): (1) the reduction in intravascular volume due to interstitial fluid leakage and (2) the direct impact of activated pancreatic enzymes, along with inflammatory mediators and cytokines. A prospective observational study of 410 patients with AP revealed that an increase in BUN was an accurate predictor of mortality (AUC: 0.842) and persistent multiorgan failure (AUC: 0.828) (Pando et al., 2021). However, Lin et al. (2017) reported that determining BUN levels 24 h after hospital admission is highly accurate for predicting SAP. In contrast, our study indicated that the initial BUN level within 24 h after admission is a good predictor of HTG-SAP. The difference in these outcomes may be attributed to the pathogenesis of HTG-AP, which needs to be confirmed by further clinical studies. PE is recognized as a strong individual predictor of SAP according to the RAC (Banks et al., 2013). In a retrospective study of 465 patients with AP from three acute pancreatitis centres, Yan et al. (2021) reported that PE volume can be a reliable predictor of SAP (*p* < 0.05), which is consistent with our results. Consequently, the RR, BUN level, and presence of PE are important for predicting the occurrence of HTG-SAP.

Early local complications, including acute peripancreatic fluid collection (APFC) and acute necrotic collection (ANC), are also components used to assess the severity of AP (Banks et al., 2013). Our study revealed that D-D level, pH, the presence of PN, and Ca^2+^ level are individual predictors of HTG-SAP. Another potential mechanism underlying how HTG-AP is aggravated involves microcirculatory disturbances, which can result in local ischaemia and damage to acinar cells (Kiss et al., 2023). An elevated concentration of chylomicrons can lead to an excess of FFAs, and capillary leakage can directly and significantly increase plasma viscosity; this elevation in viscosity may result in impaired microcirculation, capillary blockage, ischaemia, haemostatic dysfunction, and acidosis, ultimately inducing ischaemic necrosis of pancreatic tissue (Kiss et al., 2023). Dysfunction in the coagulation system may result in microcirculatory collapse and failure across multiple organs, both within and beyond the pancreas, thereby increasing the mortality rate associated with AP (Gui et al., 2023). A retrospective study of 189 patients with HTG-AP revealed that the AUC of the D-D level for the prediction of HTG-SAP was 0.753 95% CI [0.680–0.826]; *P* < 0.001); therefor, the D-D level may serve as a sensitive early prognostic indicator for HTG-SAP (Gou, Yao & Cao, 2023). Metabolic acidosis (plasma pH less than 7.35) can occur in patients with AP due to several factors, including the buildup of lactic acid caused by shock, kidney dysfunction, or, in the later stages of the disease, the loss of bicarbonate-rich pancreatic secretions due to pancreatic duct disruption. A meta-analysis including thirteen studies with 2,311 patients with AP indicated that patients with AP who exhibited reduced blood pH or base excess experienced significantly greater mortality rates and increased severity scores (Rumbus et al., 2018). In a prospective study, Sharma et al. (2014) reported that patients with AP patients with a pH less than 7.35 presented a greater incidence of organ failure and required more frequent interventions, indicating that pH is a useful early marker for predicting adverse outcomes in patients with AP. PN is a consistent prognostic factor in AP, serving as an indicator of local complications according to the RAC (Banks et al., 2013). In a retrospective study, Fu et al. (2021) demonstrated that the extrapancreatic necrosis volume could be a valuable indicator for predicting poor outcomes in patients with AP. The necrosis of pancreatic and peripancreatic fat can lead to the autodigestion of mesenteric fat. During this process, Ca^2+^ binds to the digested fat to form calcium soaps, resulting in hypocalcaemia (Kiss et al., 2023). Yu et al. (2019) reported that the Ca^2+^ levels in patients with HTG-SAP were significantly lower than those in patients with SAP of other aetiologies, with values of 1.69 (95% CI [1.46–1.91]) *versus* 2.1 (95% CI [1.93–2.23]) (*P* < 0.001), confirming that a lower calcium level is correlated with an increased risk of HTG-SAP Additionally, established prognostic system models for AP, such as the Ranson score and the Glasgow score (JSS), also include Ca^2+^ levels in their scoring systems. Therefore, the use of Ca^2+^ as a prognostic indicator in the HTG-SAP model is reasonable and logical. Overall, these four indicators reflect the pathophysiological process of pancreatic ischaemic necrosis and can effectively predict the severity of HTG-AP.

In this study, a simple and accurate nomogram model was developed and validated to predict the occurrence of HTG-SAP using variables that are easily accessible within the first 24 h of admission. The performance of nomogram was evaluated based on its discrimination, calibration, and clinical applicability, and satisfactory results were obtained upon verification. Additionally, this nomogram model provides important insights for guiding treatment decisions for high-risk patients with HTG-SAP. We compared the predictive performance of various scoring systems, including the BISAP, SOFA and MCTSI, with that of our newly developed nomogram. The AUC value of the new HTG-SAP model was 0.960 (95% CI [0.908–0.966], *P* < 0.000), which was greater than those of the BISAP, SOFA and MCTSI (AUCs: 0.782, 0.784, and 0.823, respectively). Another major strength of our prediction model is its rapid clinical applicability. Unlike established scores such as SOFA, which requires 24-hour data, or MCTSI, which may require imaging assessment at 48–72 h, our model utilizes variables (*e.g.*, RR, D-dimer, BUN, Ca^2^^+^, pH, and initial CT) that are routinely available within hours of admission (Supplemental File 4). This allows for early risk stratification within the critical initial 24 h, enabling timely intervention for high-risk patients with HTG-SAP and potentially improving outcomes. The results demonstrate that our nomogram model has superior predictive ability and greater clinical utility compared with those of other established scoring systems.

### Limitations

It is important to recognize the limitations of this study. First, this was a retrospective study, and the patients’ subjective symptoms (including the onset time of abdominal pain and mental changes) could only be judged by medical records, introducing information and selection bias. Second, this was only a single-centre study, and the characteristics of HTG-AP are significantly influenced by factors such as region, race, and lifestyle habits. Third, to consider the potential impact of PS on the results, assessing the pancreatic fat content prior to the onset of pancreatitis is prefarable; however, it is uncommon for healthy individuals to undergo CT scans. Finally, our prediction model was developed using data obtained at initial admission and did not adjust for the potential influence of postadmission therapeutic interventions, such as lipid-lowering therapy, fluid resuscitation volume, and nutritional support. Furthermore, although the CT acquisition parameters remained consistent throughout the study, the evolution of clinical guidelines over the five-year period represents another potential, unmeasured confounder. The omission of these dynamic clinical and treatment factors may affect the generalizability of the model to settings with varying practices. Future studies should aim to integrate these variables to enhance the model’s validity and clinical utility. To this end, further optimization and validation of the model are needed in a large-scale, multicentre, prospective cohort study.

## Conclusion

In summary, our study confirmed that RR, D-D level, BUN level, Ca^2+^ level, pH, and the presence of PN, PE and PS are independent predictors of HTG-SAP. The prediction model developed based on these predictors has greater consistency and practicability in predicting HTG-SAP than the BISAP, MCTSI and SOFA scores do.

## Supplemental Information

10.7717/peerj.20607/supp-1 Supplemental Information 1 Data for training The raw data of all included patients in the model training cohort, including baseline characteristics and clinical parameters.

10.7717/peerj.20607/supp-2 Supplemental Information 2 Data for validation The complete raw data of all enrolled patients in the model validation cohort, including both demographic characteristics and clinical parameters.

10.7717/peerj.20607/supp-3 Supplemental Information 3 The Value_PS data

10.7717/peerj.20607/supp-4 Supplemental Information 4 R programming code The R programming code utilized in this study encompasses:
Univariate and multivariate analyses, Predictive model development and validation, Visualization outputs including: Nomograms, Decision curve analysis (DCA) plots, Calibration curves.

10.7717/peerj.20607/supp-5 Supplemental Information 5 Codebook In the raw data, the PS was derived through indirect calculation and transformation

10.7717/peerj.20607/supp-6 Supplemental Information 6 Time to variable availability: prediction model *vs.* scoring systems Summary and comparison of the estimated “time to availability” for each predictor variable in our model alongside those required for other common scoring systems (SOFA, BISAP, and MCTSI), and provides a clear, at-a-glance overview of the practical timelines involved in data acquisition.

10.7717/peerj.20607/supp-7 Supplemental Information 7 STROBE checklist

## Abbreviations

HTG-AP: hypertriglyceridaemic acute pancreatitis
HTG-SAP: hypertriglyceridaemic severe acute pancreatitis
PS: pancreatic steatosis
HTG-NSAP: hypertriglyceridaemic nonsevere acute pancreatitis
FFAs: free fatty acids
T2DM: type 2 diabetes mellitus
HTN: hypertension
CAD: coronary heart disease
T: temperature
HR: heart rate
RR: respiratory rate
MBP: mean blood pressure
De: delirium
SIRS: systemic inflammatory response syndrome
SOFA: Sequential Organ Failure Assessment
MCTSI: modified CT Severity Index
BISAP: Bedside Index for Severity in Acute Pancreatitis
CRP: C-reactive protein
WBC: white blood cell
N: neutrophil
NR: neutrophil ratio
M: monocyte
L: lymphocyte
HCT: haematocrit
PLT: platelet
APTT: activated partial thromboplastin time
PT: prothrombin time
INR: international normalized ratio of coagulation
FIB: fibrinogen
D-D: D-dimer
LPS: lipase
AMY: amylase
PCT: procalcitonin
ALB: albumin
TB: total bilirubin
BUN: blood urea nitrogen
Cr: creatinine
Ca^2+^: calcium
GLU: glucose
LAC: lactic acid
TC: total cholesterol
TG: triglyceride
LDL: low-density lipoprotein
PH: potential of hydrogen
PaCO2: partial pressure of carbon dioxide
PaO2: partial pressure of oxygen
FiO2: fraction of inspired oxygen
PN: pancreatic necrosis
PE: Pleural effusion
AE: abdominal effusion
MCT_L: mean of CT scan value of the liver
MCT_S: mean of CT scan value of the spleen
MCT_P: mean of CT scan value of the pancreas
*β*: Regression coefficient
Pre-HTG-SAP: predictive model for hypertriglyceridaemic severe acute pancreatitis
AUC: area under the curve
DCA: decision curve analysis

## Additional Information and Declarations

### Data Availability

The following information was supplied regarding data availability: The datasets are available in the Supplemental Files.
