# Supplementary material for: Development and validation of an early prediction model for hypertriglyceridaemic severe acute pancreatitis: a retrospective study
Source: PeerJ. 2026 Jan 20;14:e20607. doi: 10.7717/peerj.20607 (PMC12829462; doi:10.7717/peerj.20607)
Supplement: Supplemental Information 5 — In the raw data, the PS was derived through indirect calculation and transformation [file peerj-14-20607-s005.docx]

In the raw data, the following categorical variable was derived through indirect calculation and transformation:

PS

Value_PS=MCT_P/MCT_S,

if Value_PS＜0.7, PS=1;

if Value_PS ≥0.7, PS=0.

The Value_PS data have been appended to the raw dataset in Additional File 3.
