# Supplementary material for: Development and validation of an early prediction model for hypertriglyceridaemic severe acute pancreatitis: a retrospective study
Source: PeerJ. 2026 Jan 20;14:e20607. doi: 10.7717/peerj.20607 (PMC12829462; doi:10.7717/peerj.20607)
Supplement: Supplemental Information 6 — Summary and comparison of the estimated “time to availability” for each predictor variable in our model alongside those required for other common scoring systems (SOFA, BISAP, and MCTSI), and provides a clear, at-a-glance overview of the practical timelines involved in data acquisition. [file peerj-14-20607-s006.docx]

| **Model** | **Time to availability** |
| --- | --- |
| Pre-HTG-SAP | Within 24 hours of admission |
| Baseline characteristic：RR | Within 1 hours of admission |
| Blood tests：D-D level, BUN level, Ca2+ level, pH level | Within 4 hours of admission |
| CT scan：the presence of PN, PE and PS | Within 12–24 hours of admission |
| SOFA | Within 24 hours of admission |
| BISAP | Within 24 hours of admission |
| MCTSI | 48-72 hours after admission |

**Time to Variable Availability: Prediction Model vs. Scoring Systems**

**Abbreviations:** RR, Respiratory rate; D-D, D-dimer; BUN, Blood urea nitrogen; Ca^2+^, Serum calcium; PH, Potential of hydrogen; PN, pancreatic necrosis; PE, Pleural effusion; PS, Pancreatic steatosis; MCTSI, Modified CT Severity Index; BISAP, Bedside Index for Severity in Acute

Pancreatitis; SOFA, Sequential Organ Failure Assessment; Pre-HTG-SAP, Predictive model for hypertriglyceridaemic severe acute pancreatitis.
